# Paracrine regulation of pancreatic cancer cell response to chemotherapy by GLI2–collagen I signaling

**DOI:** 10.1016/j.jbc.2025.110311

**Published:** 2025-05-29

**Authors:** Renzo E. Vera, Maite G. Fernandez-Barrena, Jose M. Falero, John Y. Kwon, Roberto A. Garza, Ashley N. Sigafoos, Matthew D. Ross, Merih Deniz Toruner, Murat Toruner, Ezequiel J. Tolosa, Luciana L. Almada, Huocong Huang, Rolf A. Brekken, Martín E. Fernandez-Zapico

**Affiliations:** 1 Schulze Center for Novel Therapeutics, Division of Oncology Research, Rochester, Minnesota, USA; 2 Department of Surgery, Hamon Center for Therapeutic Oncology Research, UT Southwestern, Dallas, Texas, USA; 3 Department of Immunology, Hamon Center for Therapeutic Oncology Research, UT Southwestern, Dallas, Texas, USA; 4 Department of Pharmacology, Hamon Center for Therapeutic Oncology Research, UT Southwestern, Dallas, Texas, USA

**Keywords:** collagen, pancreatic cancer, tumor microenvironment, GLI2, fibroblast

## Abstract

Despite the well-described role of noncellular components of the tumor microenvironment (TME) in regulating tumor growth, the molecular events dictating expression and biological functions of key components of the TME remain elusive. Here, using pancreatic cancer (PC) models, we describe a novel mechanism through which the zinc finger transcription factor GLI2 in cancer-associated fibroblasts (CAFs) induces expression of *COL1A1*, which is a major component of type I collagen, the most abundant collagen variant in the tumor milieu. Bulk and single-nuclei RNA-Seq showed that *GLI2* expression in CAF strongly correlates with *COL1A1* expression levels, fibrosis, and CAF activation. Chromatin immunoprecipitation–quantitative PCR and expression studies of the PC matrisome identified *COL1A1* as the direct target of GLI2 in CAFs. We also provide evidence that GLI2 is an effector that mediates *COL1A1* induction by transforming growth factor β1. RNA-Seq analysis of PC cells treated with type I collagen revealed enrichment of chemotherapeutic gene expression profiles, which includes irinotecan resistance signature. Viability studies confirmed that type I collagen promotes irinotecan resistance in PC cells. Altogether, our results uncover a novel role for the transforming growth factor β1–GLI2 axis within CAFs to modulate type I collagen expression and promote chemoresistance in PC cells. Together, our findings help increase the understanding of the complex molecular network operating in the TME.

Despite decades of research, pancreatic cancer (PC) remains a fatal disease and is projected to become the second leading cause of cancer-related death in the United States by 2040 (1). Although some advancements in frontline therapies for PC have been made over the last several years (2, 3, 4), chemoresistance and a paucity of effective molecular targets remain significant barriers to progress in the management of this deadly disease, which has a 5-year overall survival rate of ∼13% (5). There is, therefore, a need for a better understanding of PC pathobiology to help identify more effective therapies for this malignancy. In recent years, numerous reports have highlighted the importance of the tumor microenvironment (TME) as a leading force in PC evolution (6, 7). The protumorigenic behavior of the TME is driven by the complex interplay between diverse cellular population (including cancer-associated fibroblasts [CAFs], immune, and endothelial cells) and the dense matrisome predominantly composed of collagens, integrins, and proteoglycans. Increasing our knowledge of the mechanisms modulating this interplay will help in understanding the function of the TME in PC biology and therapeutics.

Here, we provide evidence for a novel mechanism in which the transcription factor GLI2 acts as a downstream effector of transforming growth factor β1 (TGFβ1) signaling in CAFs to drive therapeutic resistance in PC cells. TGFβ is a well-established cascade regulating PC TME by controlling noncellular structures and cellular dynamics (8, 9, 10, 11, 12, 13). TGFβ signaling is controlled by three major ligands, TGFβ1 is the most upregulated in PC, and it has been reported to contribute with disease progression and correlates with lower survival (8, 9). Our association studies demonstrated that the expression of GLI2 and type I collagen is positively correlated in PC samples with high grade of fibrosis, a common feature of this malignancy. At the same time, we probed that the activation of CAFs was associated with higher expression of *GLI2* and *COL1A1*, a major component of type I collagen (14, 15, 16, 17). Harnessing a combination of chromatin immunoprecipitation–quantitative PCR (ChIP–qPCR), luciferase, and expression studies, we demonstrated that *COL1A1* is a direct transcriptional target of GLI2 downstream of TGFβ1. In addition, we have shown an enrichment in gene signatures associated with irinotecan resistance in PC cells treated with collagen I. Further studies confirmed the resistance of PC cells to SN38, the active metabolite of irinotecan, upon incubation with type I collagen. These results underscore the molecular feedback between CAFs and PC cells, which involves regulation of the TME and response to chemotherapeutic agents.

## Results

### GLI2 expression correlates with the levels of fibrosis in PC patients

**Figure 1:**
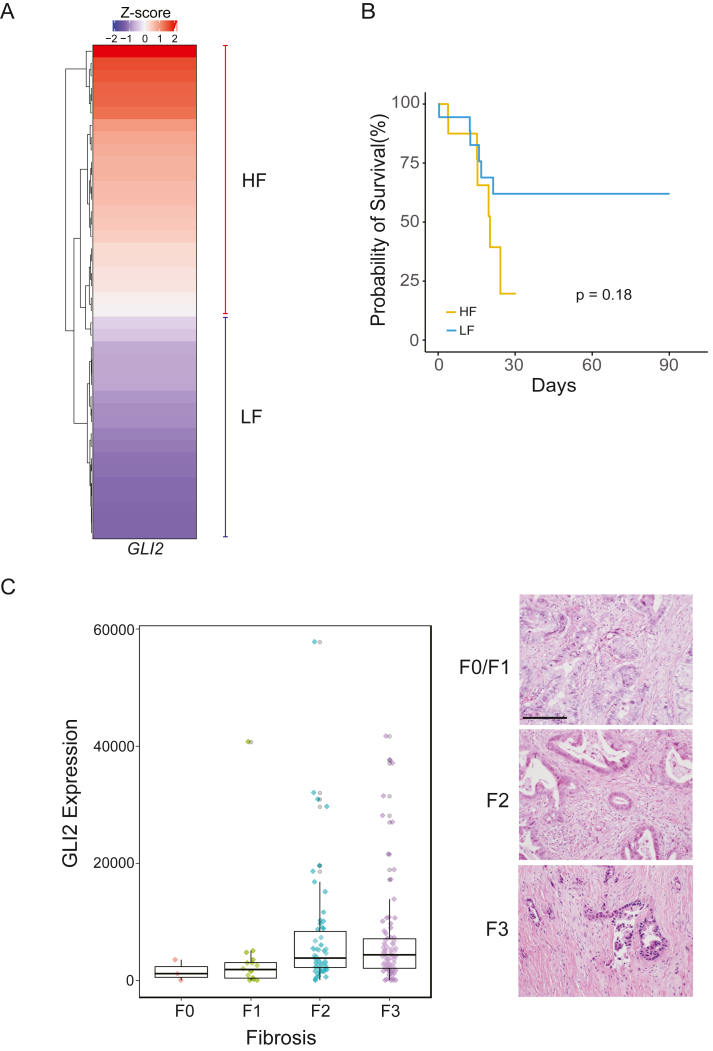
**PC patients with high levels of fibrosis are positively correlated with GLI2 expression**. *A,* heatmap showing the Z-score for GLI2 in PC patients (n = 40) classified with high fibrosis (HF) or low fibrosis (LF) according to the TME Cell Population-counter (MCP-counter) method from bulk RNA-Seq data (Pan-Cancer atlas, 2018). *B,* Kaplan–Meier curve depicting survival in HF and LF patients (total n = 36, log-rank test, *p* = 0.18). *C, left,* evaluation of *GLI2* expression and fibrosis level (from F0 to F3) in PC patient samples (n = 158). *Right,* representative images from H&E-stained tissues with different grades of fibrosis. Scale bar represents 100 μm. PC, pancreatic cancer; TME, tumor microenvironment.

First, we examined the expression of *GLI2* in relation to the grade of CAF tumor infiltration, an indicator of levels of fibrosis, in The Cancer Genome Atlas samples (Pan-Cancer atlas, 2018). Employing the Microenvironment Cell Population-counter (MCP-counter) method (18), tumor samples were scored according to the grade of CAF infiltration. We observed that more than 90% of the patients with the highest fibrosis score displayed a high Z-score for *GLI2* expression in comparison to patients with low fibrosis (LF) score (Fig. 1*A*). Survival curves showed that patients with high fibrosis (HF)/high *GLI2* expression (HF group) tend to have less survival probability than LF/low *GLI2* patients (LF group) (Fig. 1*B*). These results were confirmed in separate cohorts (19, 20) showing that tumors with HF display increased levels of *GLI2* (Fig. S1, *A* and *B*). Next, we validated these results in our institutional cohort of PC patients. Tissue samples were classified based on the grade of fibrosis (F0–F3) (Fig. 1*C*, *right panel*). Using qPCR, the expression of GLI2 was measured. Results show increased levels of this transcription factor in samples with high-grade fibrosis (F3) in comparison to low-grade fibrosis (F0/F1) (Fig. 1*C*). Finally, to define the cellular compartment associated with increased *GLI2* expression and candidate profibrotic function, we used single-nucleus RNA-Seq (snRNA-Seq) data from publicly available sources (21). The analysis provides evidence that the transcriptional activity of *GLI2* was mainly associated with the fibroblast compartment (Fig. S1, *C* and *D*). Thus, employing multiple approaches, we confirmed a positive correlation between fibrosis and GLI2, presenting the fibroblast compartment in the tumor stroma as the major source of GLI2 activity.

### GLI2 is an effector of TGF**β**1 to induce type I collagen by CAFs

**Figure 2:**
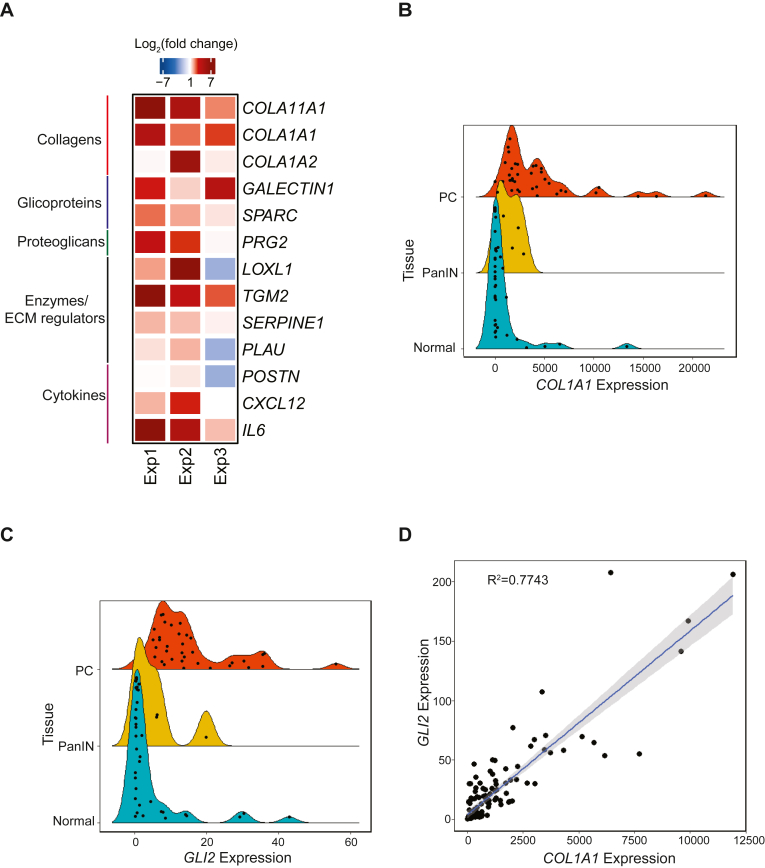
***GLI2* and *COL1A1* expression are associated *in vitro* and *in vivo*.***A,* heatmap showing the relative expression levels of matrisome genes under *GLI2* overexpression conditions. HPSCs were transfected with pCMV-GLI2 or pCMV empty vector. Twenty-four hours later, RNA was extracted and the expression of selected matrisome genes was evaluated by qPCR and referred to the control (n = 3). *B,* comparative expression of *COL1A1* in samples categorized as normal pancreas, premalignant (PanIN), and pancreatic cancer (PC) tissues. Extraction of RNA was performed in patient's tissue samples. qPCR was employed for gene expression (n = 70). *C,* comparative expression of *GLI2* in samples categorized described above. qPCR was employed for gene expression (n = 70). *D,* linear regression analysis for *GLI2* and *COL1A1* taking the expression analysis performed in *B* and *C*. *R*^2^ = 0.77. HPSC, human pancreatic stellate cell; PanIN, pancreatic intraepithelial neoplasia; qPCR, quantitative PCR.

Given the aforementioned results, we hypothesize that GLI2 may regulate the transcription of key components of PC extracellular matrix. We curated a list of top molecules reported to be upregulated in PC matrisome (22, 23, 24) and analyzed using snRNA-Seq data. The analysis reveals that *GLI2* and the collagen genes *COL11A1*, *COL1A1*, and *COL1A2* are highly enriched in fibroblasts (CAFs) compared with other cellular compartments in the TME (Fig. S2, *A*–*C*). Also, we observed enriched expression in the fibroblast compartment for other components of the matrisome, such as SPARC, SERPINE1, and POSTN (Fig. S2, *D*–*F*), with no differences in the expression levels of LGALS3, LOXL1, PLAU, PRG2, TGM2, CXCL12, and IL6 **(**Fig. S2, *G–M*). These results raise the possibility that GLI2 may promote the transcription of collagen genes. To define this association between GLI2 and critical PC-related matrisomal proteins, we overexpressed GLI2 in a PC CAF cell line (human pancreatic stellate cell [HPSC]). qPCR results showed that CAFs harboring high GLI2 expression enhanced the expression of these targeted extracellular matrix components, including *COL1A1* and *COL11A1*, compared with their control counterparts (Fig. 2*A*). As type I collagen is one of the main components in the TME (14, 15, 16, 17), we focused on studying the functional interplay between GLI2 and *COL1A1*. In our patient cohort, we found higher levels for *COL1A1* and *GLI2* in PC in comparison to healthy and pancreatic intraepithelial neoplasia tissues (Fig. 2, *B* and *C*) and a positive correlation between *GLI2* and *COL1A1* in tumors (Fig. 2*D*).

**Figure 3:**
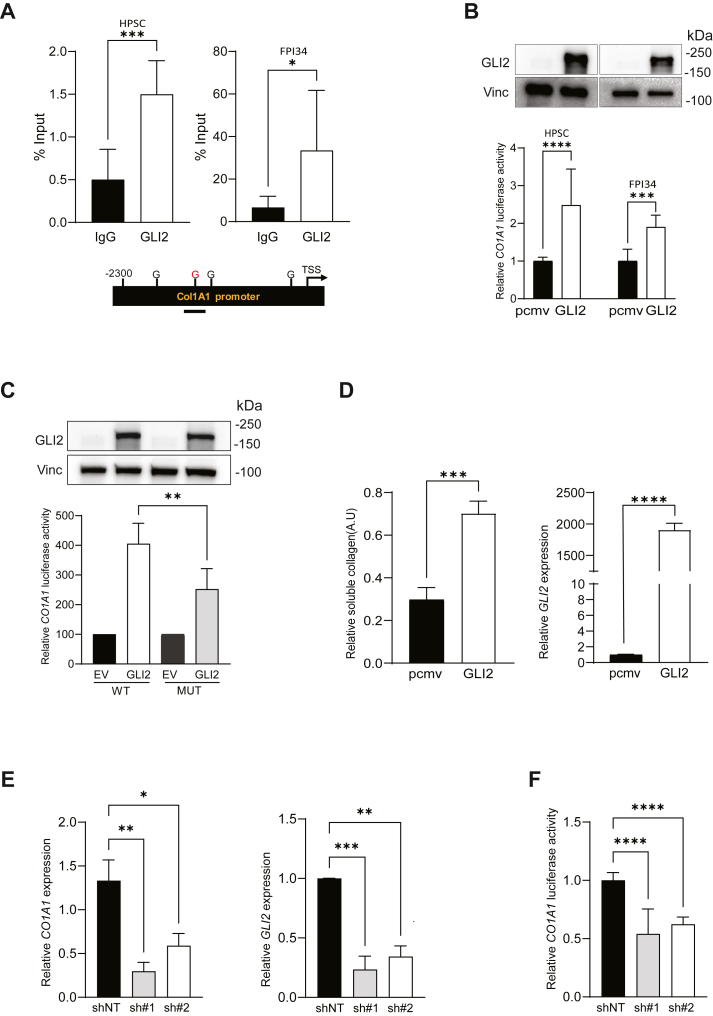
**COL1A1 is a direct target of GLI2 transcription factor in CAFs**. *A, top panel,* ChIP assay for HPSC and FPI34 fibroblasts showing the endogenous binding (% of Input) of GLI2 to the *COL1A1* promoter. Normal human immunoglobulin G (IgG) was taken as control. *Bottom panel,* schematic representation of the *COL1A1* gene promoter region with the four predicted GLI2-binding sites. *Line* represents the promoter region analyzed in the ChIP assay. *B, top panel,* Western blots showing the overexpression of GLI2. HPSC and FPI34 cells were transfected with pCMV-GLI2 or pCMV empty vector (EV). Twenty-four hours later, protein lysates were collected and analyzed. Vinculin was used as loading control. *Bottom panel,* luciferase reporter assay in HPSC and FPI34 fibroblast showing *COL1A1* promoter activation under GLI2 overexpression conditions. *C, top panel,* Western blots showing the expression of GLI2. HPSCs were transfected with pMV-GLI2 (GLI2) or pMV EV in combination with either the WT or mutant (MUT) COL1A1 reporter. Twenty-four hours later, protein lysates were collected, quantified, and analyzed. Vinculin was used as loading control. *Bottom panel,* luciferase reporter assay in HPSC showing WT or MUT promoter activation under GLI2 or EV overexpression conditions. *D, right,* relative soluble type I collagen levels determined by Sircol assay in supernatants from GLI2-overexpressing fibroblasts. *Left,* qPCR assay showing the relative *GLI2* mRNA expression in fibroblasts used to analyze the secretion of soluble collagen I. *E, left,* relative *COL1A1* mRNA expression in HPSC fibroblasts treated with two shRNAs (sh#1 and sh#2) targeting *GLI2* or the nontargeting control (shNT). *Right,* GLI2 expression in HPSCs transfected with aforementioned shRNAs. RNA was collected 24 h post treatment, and qPCR was performed. *F,* luciferase reporter assay for *COL1A1* promoter under *GLI2* silencing conditions (sh#1 and sh#2) or control (shNT). Results are expressed as means ± SD. Statistical significance, ∗*p* < 0.05; ∗∗*p* < 0.01; ∗∗∗*p* < 0.001; and ∗∗∗∗*p* < 0.0001. CAF, cancer-associated fibroblast; ChIP, chromatin immunoprecipitation; G, GLI binding site; qPCR, quantitative PCR; TSS, transcription start site.

Next, we sought to define if *COL1A1* is a direct target of GLI2. Bioinformatic analysis showed that the *COL1A1* promoter has four potential GLI consensus DNA-binding sites (5′-CCACCCAG-3′) (Fig. 3*A*, *lower panel*). ChIP–qPCR assays using GLI2 antibody in two different CAF cell lines demonstrated that consistently GLI2 occupies one of these binding sites on the *COL1A1* promoter (Fig. 3*A*, *upper panel*). Using a *COL1A1* promoter luciferase reporter construct, we show that the overexpression of GLI2 in HPSC and FPI34 cells increased *COL1A1* promoter activity (Fig. 3*B*, *lower panel*). The level of expression of GLI2 was determined by Western blot (Fig. 3*B*, *upper panel*). Furthermore, we sought to validate the importance of the identified GLI2 binding site in *COL1A1* promoter activity. Luciferase assay was performed employing a mutant construct carrying a deletion of this GLI2 binding site previously defined by ChIP–qPCR (Fig. 3*A*). The results showed a reduction in activity of the mutant promoter compared with the WT counterpart in CAFs overexpressing GLI2 (Fig. 3*C*, *lower panel*). Equal levels of GLI2 expression were confirmed by Western blot (Fig. 3*C*, *upper panel*). In addition, we evaluated whether forced GLI2 expression increased the levels of soluble type I collagen in HPSCs. The level of *GLI2* expression was checked by qPCR (Fig. 3*D*, *right panel*). Cell culture supernatants recovered from these experimental groups showed a significant increase in the concentration of soluble type I collagen in the conditioned media from GLI2-overexpressing fibroblasts (Fig. 3*D*, *left panel*). Next, employing two independent shRNAs in HPSCs, we showed that the knockdown of *GLI2* correlated with a reduction in *COL1A1* expression (Fig. 3*E*, *left panel*) and promoter activity (Fig. 3*F*). GLI2 knockdown was confirmed by qPCR (Fig. 3*E*, *right panel*).

**Figure 4:**
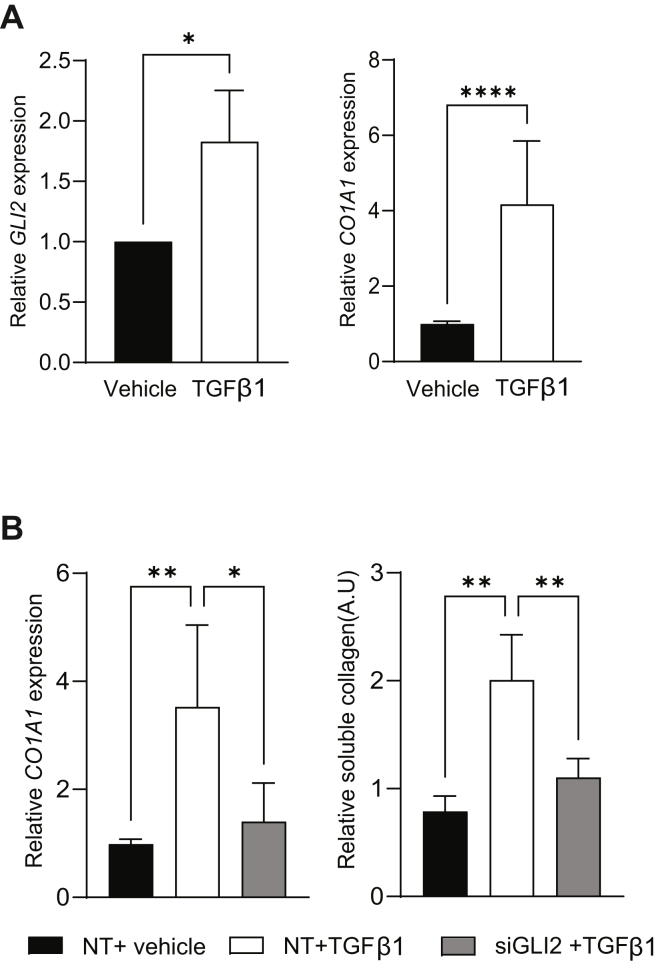
**TGFβ1 regulates the secretion of soluble type I collagen through the activity of GLI2**. *A, left,* relative *GLI2* mRNA expression in HPSC fibroblast treated with TGFβ1 or vehicle (control). *Right,* relative *COL1A1* mRNA expression in HPSC fibroblasts treated with TGFβ1 or vehicle (control). HPSCs were treated with 20 ng/ml TGFβ1 over 72 h. RNA was collected, and qPCR was performed (n = 3). *B, left,* effect of GLI2 silencing on *COL1A1* expression triggered by TGFβ1. HPSCs were incubated with siRNA targeting GLI2 (siGLI2) or nontargeting control (NT). After 48 h, cells from each group were incubated with TGFβ1 or vehicle over 72 h. RNA was collected, and qPCR was assayed (n = 3). *Right,* effect of GLI2 silencing on soluble type I collagen secretion triggered by TGFβ1. HPSCs were treated with siGLI2 or NT. After 48 h, cells from each group were incubated with TGFβ1 or vehicle over 72 h. Supernatants from these cell cultures were harvested, and soluble type I collagen was measured by Sircol assay (n = 3). Results are expressed as means ± SD. Statistical significance, ∗*p* < 0.05; ∗∗*p* < 0.01; and ∗∗∗∗*p* < 0.0001. HPSC, human pancreatic stellate cell; qPCR, quantitative PCR; TGFβ1, transforming growth factor β1.

Given the fact that TGFβ1 is a key regulator of PC TME and fibroblast activity (8, 9, 10, 11, 12, 13, 25, 26) and is enriched in the fibroblast compartment (Fig. S2*N*), we evaluated whether TGFβ1-induced *COL1A1* expression depends on GLI2 activity in our CAF models. To this end**,** CAF HPSCs were treated with 20 ng/ml of TGFβ1 for 48 h. Expression analysis showed induction of *GLI2* and *COL1A1* expression upon TGFβ1 stimulation (Fig. 4*A*). Similar results were seen in a TGFβ1-responsive fibroblast line (MRC-5). Treatment with TGFβ1 (4 ng/ml) for 48 h showed strong induction of the collagen synthesis–related genes *COL1A1* and *COL1A2* as well as *GLI2* (Fig. S3*A*). As expected, TGFβ1 induced the expression of classic CAF activation markers, such as *FAP*, *ACTA2*, *CXCL12*, and *IL6* (Fig. S3*B*). Interestingly, siRNA-based knockdown of *GLI2* (Fig. S3*C*) abrogated the mRNA induction of *COL1A1* by TGFβ1 (Fig. 4*B*, *left panel*). Finally, we demonstrated that the GLI2 knockdown also led to reduced soluble collagen levels (Fig. 4*B*, *right panel*). Our results demonstrated that *COL1A1* is a novel direct transcriptional target of GLI2 downstream of TGFβ1 signaling.

### Type I collagen promotes irinotecan resistance in PC cells

**Figure 5:**
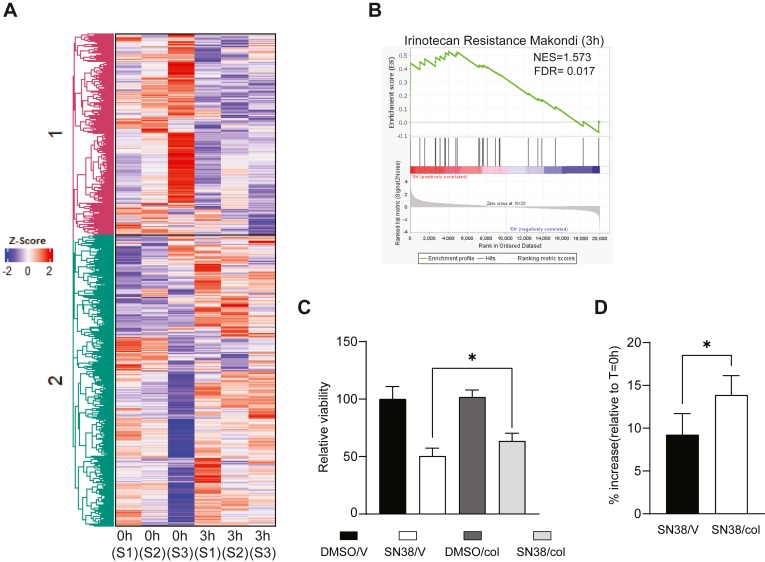
**Soluble type I collagen induces chemotherapy resistance in PC cells.***A,* heatmap showing the Z-score and the clustering from RNA-Seq. PANC-1 cells were treated with type I collagen. Samples were collected 3 h post treatment. RNA was extracted and employed for RNA-Seq analysis (n = 3). *B,* gene set enrichment analysis (GSEA) showing enrichment in pathways associated with irinotecan resistance. *C,* viability analysis of MIA Paca-2 cells. Cell cultures were incubated 24 h with dimethyl sulfoxide (DMSO) or 4 mM of the active metabolite of irinotecan (SN38) in combination with either 50 μg/ml of type I collagen (col) or vehicle (V). After treatment, the viability was determined by MTT assay (n = 3). *D,* cell counting assay of MIA Paca-2 cells. Cell cultures were incubated 24 h with either DMSO or 4 mM of SN38 in the presence of 50 μg/ml of col or V. The cell number was determined using Celigo Imager (n = 3). Results are expressed as means ± SD. Statistical significance, ∗*p* < 0.05. MTT, 3-(4,5-Dimethylthiazol-2-yl)-2,5-diphenyltetrazolium bromide; PC, pancreatic cancer.

To shed light on the biological consequences of these increased levels of collagen, PANC-1 cells were stimulated with 10 μg/ml of type I collagen, and RNA was collected 3 h and 24 h after treatment for RNA-Seq analysis. Unsupervised clustering revealed that as soon as 3 h post treatment, there is differential gene expression between control and type I collagen-treated samples (Fig. 5*A*). The upregulated genes were related to highly aggressive, tumor-promoting pathways, such as inflammatory response, early growth response signaling, and oncogenic cytokine–receptor interaction (Fig. S4*A*–*C*) (27, 28, 29). Interestingly, gene set enrichment analysis (GSEA) shows that genes associated with irinotecan resistance were enriched after type I collagen stimulation (Fig. 5*B*, Table S1). Similar results were obtained from samples treated for 24 h with type I collagen (Fig. S4*D*). Taking into consideration these results, we decided to determine if type I collagen can impact PC cell response to SN38. MIA Paca-2 cells were treated with 4 μM of SN38 or dimethyl sulfoxide (DMSO) over 24 h in combination with 50 μg/ml type I collagen (Col) or vehicle (V). 3-(4,5-Dimethylthiazol-2-yl)-2,5-diphenyltetrazolium bromide (MTT) assays revealed that these tumor cells showed sensitivity to SN38. However, type I collagen–treated cells exhibited higher viability compared with the control-treated cells (Fig. 5*C*). Similar results were observed in PANC-1 cells (Fig. S4*E*). To validate our observations, a cell counting assay was performed on MIA Paca-2 cells after the treatments described previously. While the presence of type I collagen in SN38-treated cells increased the cell count (Fig. 5*D*). Interestingly, type I collagen has no effect on the cell count in the control vehicle group (Fig. S4*F*). Altogether, our results highlight a novel role for soluble type I collagen in modulating PC cell sensitivity to irinotecan.

## Discussion

PC is characterized by a high level of fibrosis. This has been considered a key feature of the PC TME promoting progression and therapeutic resistance (30, 31). Thus, significant interest has been raised in understanding the mechanisms contributing to the expression of the components of the matrisome in PC TME. CAFs are one of the primary cell types that generate extracellular proteins in the tumor milieu (32, 33, 34). Type I collagen is the main component of this PC matrisome, impacting tumor cell responses through its insoluble and soluble variants (23, 35). There is extensive literature describing the contribution of insoluble collagen meshes modulating mechanotransduction and reducing the penetration of chemotherapeutic drugs (36, 37, 38, 39). However, the function of soluble type I collagen in tumor biology has remained poorly understood. Thus, research is needed to define the molecular mechanisms leading to the secretion of type I collagen by CAFs and its biological consequences.

Previous research has shown the expansion of GLI-expressing fibroblasts during stroma remodeling associated with the transition from pancreatic intraepithelial neoplasia to PC (40). In addition, abrogation of HH signaling modified the proportion of different CAF subtypes in the TME revealing the impact of the HH–GLI signaling on the fate of CAF phenotype (41). In previous reports, we showed the role of GLI transcription factors (mainly GLI1) in CAFs to regulate the secretion of CXCL12 to promote tumor cell migration (42). Here, we provided evidence for a new role of another member of the GLI family of transcription factors, GLI2, in the regulation of matrisome genes, specifically COL1A1, a major component of type I collagen. Combining bulk and snRNA-Seq, together with histological analysis, we showed that high GLI2 expression correlated with increased expression of *COL1A*1 and grade of fibrosis. This correlation guided us to investigate a direct regulation of *COL1A1* by GLI2. Employing a combination of chromatin binding assays, together with functional studies, we demonstrate that *COL1A1* is a novel target for GLI2 in CAFs. Moreover, we supported that this novel axis is controlled upstream by TGFβ1, a well-known regulator of CAF phenotype (43, 44, 45, 46).

Using bulk RNA-Seq, we defined changes in the transcriptional program of PC cells upon type I collagen treatment. We observed modifications in the transcriptional profile as soon as 3 h post treatment. GSEA revealed an increase in the expression of cell death regulators and proinflammatory cytokines. These results are in concordance with previous studies showing specific patterns of cytokines in tissue and plasma from PC patients (47, 48). Interestingly, our analysis also revealed enrichment in gene sets associated with chemotherapeutic resistance including irinotecan. PC therapeutic options are limited; however, in recent years, the Food and Drug Administration approved irinotecan liposome as a first-line treatment for PC patients with metastatic disease (NCT04083235). Irinotecan is also being used in PC patients in combination with other agents, such as oxaliplatin and 5-fluorouracil (49, 50). Thus, we decided to determine the impact of type I collagen on PC response to irinotecan. Viability assays showed that the presence of type I collagen reduced the effectiveness of irinotecan. Furthermore, our results indicate that mainly type I collagen secreted by fibroblasts can drive this chemoresistance phenotype. This is relevant, given the fact that PC cells have shown resistance to 5-fluorouracil, oxaliplatin, and irinotecan (51, 52, 53, 54), separately.

Together, our study supports the concept that the fibrotic stroma enriched in CAF-expressing GLI2 is shaping, through the secretion of soluble type I collagen, a chemoresistant TME. This knowledge will help the design of new treatments or modify existing ones based on the TME composition and dynamics to improve patient outcomes.

## Experimental procedures

### Cell lines and culture conditions

PANC-1 (CRL-1469) and MIA Paca-2 (CRL-1420) PC cells were obtained from the American Type Culture Collection. HPSCs were a gift from Dr Rosa Hwang (Department of Breast Surgical Oncology, Division of Surgery, MD Anderson Cancer Center, Houston, TX). FPI34 human pancreatic CAFs were a gift from Dr Kenneth Olive (Columbia University Medical Center, New York, NY). All the cell lines were cultured in Dulbecco's modified Eagle's medium (DMEM) (10-013-CV; Corning) supplemented with 10% fetal bovine serum (FBS; 35-010-CV; Corning). Normal fibroblast cell line (CCL-171, MRC-5) was obtained from the American Type Culture Collection and cultured in Eagle's minimum essential medium (10-010-CV; Corning) supplemented with 10% of FBS. Cell lines were incubated using standard conditions of 37 °C and 5% CO_2_.

### TGF**β**1 treatment

For TGFβ1 treatment, 20 ng/ml (HPSC) or 4 ng/ml (MRC-5) TGFβ1 recombinant ligand (240-B-002/CF; R&D Systems) was added to cells in complete media. Samples were collected 24 h after treatment for expression studies. For detection of soluble type I collagen, HPSCs were maintained under treatment for a period of 72 h.

### Plasmids, siRNAs, and transfection

For GLI2 knockdown, siRNA directed against GLI2, siGLI2 (S103063641), and nontargeting control (1027281) were purchased from Qiagen. FPI34 and HPSCs were transfected using RNAiMax (13778-500; Qiagen) per the manufacturer's instructions.

The GLI2 expression construct was kindly provided by Dr Chi-chung Hui (Research Institute, Toronto, Ontario, Canada). The *COL1A1* promoter luciferase reporter construct was provided by Dr Cordula Büttner (Institute of Immunology, Medical Faculty, Technical University Dresden, Dresden, Germany).

To delete Gli binding site in *COL1A1* promoter luciferase plasmid, primers were designed and obtained by IDT to create Gli binding site deletion (ccacccag) ∼1.2 kb upstream of TSS plasmid (pGL3-Col1a1-GliBSDel-Luc). Fragments to remove the Gli site were amplified using Terra Direct PCR Red Dye Premix kit (TakaRa; catalog no.: 639286) along with the following primers: Gib-Col1a1-F1 5′-CCTGCTGTCCTTCGGGTCCCCA-3′/Gib-Col1a1-del-R1 5′-CAGTCAAACCAGAGAGAACAGGGAGGGCA-3′ (316 bp) and Gib-Col1a1-del-F1 5′-TAGCTAAGTGCCCTCCCTGTTCTCTCTG-3′/Gib-Col1a1-R1 5′-GCCTTCCTCCAAACCCTAGGCAT-3′ (367 bp). WT pGL3-Col1a1-Luc plasmid was opened using restriction enzymes KflI and AvrII, vector backbone (6625 bp) was gel purified along with PCR fragments for Gli binding site deletion. Gibson Assembly Cloning Kit (NEB; catalog no.: E5510S) protocol was followed according to the manufacturer to create pGL3-Col1a1-GliBSDel-Luc plasmid. Sanger sequencing was performed to confirm plasmids and proper deletion of Gli binding site.

In experiments where GLI2 was overexpressed, cells were transfected using X-TremeGENE HP DNA Transfection Reagent (06366546001; Roche), employed in the transfection procedure with a ratio of 1 μg of DNA:2.5 μl of reagent. The plasmid pMEV-2HA-Gli2-FL was created by ligation of the vector pMEV-2HA (4326 bp) and human Gli2-FL (4707 bp) using restriction sites EcoRI and XbaI.

shRNAs targeting GLI2 were purchased from Origene Technologies using plasmid vector pGFP-V-RS. The following shRNA targeting sequences were used: shGLI2(1), 5′-CCACGGAAAGCACTGGCTTCTCTGACAAC-3′; shGLI2(2), 5-TCACCAAGAAGCAGCGCAATGACGTGCAC-3.

### Luciferase reporter assay

FPI34 and HPSCs were grown and transfected in triplicate in 6-well plates as previously described. Samples were harvested and prepared per the manufacturer protocol (E1941; Dual-Luciferase Reporter Assay System; Promega). To control intersample variations in transfection efficiency, the total protein concentration of samples was quantified per well (23227; Thermo Fisher Scientific), and luciferase readouts were normalized to the respective protein concentration. Relative luciferase activity represents luciferase readout/protein concentration normalized to control cells within each experiment.

### Reverse transcription and qPCR

Total RNA was extracted from cultured cells using TRIzol reagent (15596018; Invitrogen) following the manufacturer's protocol. Two micrograms of total RNA were reverse-transcribed using a High-Capacity Complementary DNA (cDNA) Reverse Transcription Kit (4368813; Thermo Fisher Scientific). A portion of the total cDNA was amplified by real-time PCR. Samples were prepared with iTaq Universal SYBR Green Supermix (1725121; Bio-Rad) and the following primers:

**Table:** 

Gene	Primers (5′→3′)
*TGFβ1*	CAAGCAGAGTACACACAGCA (sense)
	GATGCTGGGCCCTCTCCAGC (antisense)
*GLI2*	AGACGACGTGGTGCAGTACATCAA (sense)
	CAGCTGCTGCATGTAGTTTACCCT (antisense)
*COL11A1*	GATTGTAGCCCGGCTGAAGA (sense)
	TTCGGGTCAATGCACACTTGT (antisense)
*COL1A1*	TGCCAGTCTACAGGCCTATCAGCA (sense)
	AGTCTGAGTTATCGGTGCTGAGTC (antisense)
*COL1A2*	CGGCAGCAGGAGGTTTCGGC (sense)
	GTCGCAGAGCCCCTGGGTCA (antisense)
*GALECTIN1*	TGCAACAGCAAGGACGGC (sense)
	CACCTCTGCAACACTTCCA (antisense)
*SPARC*	GGCGAGTTTGAGAAGGTGTG (sense)
	TTTGCAAGGCCCGATGTAGT (antisense)
*PRG2*	ACTTTGCATACTGGGCTGCT (sense)
	CAGTGGCCTCCTCGGGTA (antisense)
*LOXL1*	TGGCTGAACTCGTCCATGCTGTG (sense)
	ACTACGATGTGCGGGTGCTACTG (antisense)
*TGM2*	GTGGAGTCGTGGATGACCAG (sense)
	CACAGCAGTACGTCCCTTCG (antisense)
*SERPINE1*	CCGCCTCTTCCACAAATCAG (sense)
	AATGTTGGTGAGGGCAGAGA (antisense)
*PLAU*	AGGCTTAACTCCAACACGCA (sense)
	CGGATCTTCAGCAAGGCAAT (antisense)
*POSTN*	GTCTTTGAGACGCTGGAAGG (sense)
	AGATCCGTGAAGGTGGTTTG (antisense)
*CXCL12*	GATTGTAGCCCGGCTGAAGA (sense)
	TTCGGGTCAATGCACACTTGT (antisense)
*IL6*	TCAGCCCTGAGAAAGGAGACA (sense)
	ACCAGGCAAGTCTCCTCATTG (antisense)
*TBP*	GGTTTGCTGCGGTAATCATGA (sense)
	CTCCTGTGCACACCATTTTCC (antisense)
*PRT*	TGGAAAAGCAAAATACAAAGCCTAAGATGA (sense)
	ATCCGCCCAAAGGGAACTGATAGTC (antisense)

qPCR was performed in a CFX384 Touch Real-Time PCR Detection System (Bio-Rad) using the following thermal protocol: 50 °C for 2 min, then 95 °C for 10 min, followed by 40 cycles of amplification (95 °C for 15 s and 60 °C for 60 s). Melting curve analysis was performed in the range of 65 to 95 °C, 0.5 °C per 5 s increments. Sample mRNA levels were normalized to their respective PRT and TBP mRNA levels. Fold change in expression was calculated using the 2ΔCp method. The iTaq Universal SYBR Green Supermix reagent (1725122; Bio-Rad) was used for amplification and detection. Heatmap representing relative normalized gene expression was generated using ComplexHeatmap package (https://CRAN.R-project.org/package=pheatmap) in RStudio (R, version 4.2.0).

Transcript expressions in patient samples were determined by qPCR using TaqMan fluorescence methodology and ABI 7900 (Applied Biosystems). Predesigned primer/probe sets for *GLI2*, *COL1A1*, and 18s rRNA expression were purchased from Applied Biosystems. Total RNA isolation was done following the protocol in the Qiagen RNeasy kit. Five micrograms of RNA were reverse transcribed using High-Capacity cDNA synthesis kit (Applied Biosystems). A portion of the total cDNA from each sample was used for qPCR analysis. All reactions including controls were performed in triplicate. The relative target gene expression was normalized to the endogenous reference gene (18s rRNA) and determined using the ΔΔCT method.

### ChIP assay

ChIP was performed as previously described (43). Briefly, FPI34 or HPSCs (∼4 × 10^6^) were crosslinked with 1% formaldehyde added to the growth medium for 10 min at room temperature. Cells were then washed and scraped with PBS, centrifuged at 800 g for 5 min at 4 °C, resuspended in cell lysis buffer, and incubated on ice for 15 min. The pellet was then resuspended in nuclear lysis buffer, following which DNA was sheared to fragments of ∼700 bp by 50 cycles of sonication (30 s of sonication followed by 30 s of rest per cycle) (Bioruptor 300; Diagenode). Samples were then immunoprecipitated using the following antibodies: samples were then immunoprecipitated using a GLI2 antibody (NB600-872; Novus Biologicals) or normal rabbit IgG (ab37415; Abcam) at 4 °C overnight. Samples were on a rotating wheel. Following immunoprecipitation, crosslinks were removed, and immunoprecipitated DNA was purified using spin columns and subsequently amplified by PCR. qPCR of ChIP products and genomic input DNA were performed using primer set for an area containing GLI binding sites in the *COL1A1* promoter. The sequences of the primers are the following: 5′-CCAACACTGAGTCCAGGTACAACT-3′ (sense) and 5′-TAGTGGGAGGCCTGTGATCATT-3′ (antisense). Quantitative SYBR PCR was performed in triplicate for each sample or control using the CFX384 Touch Real-Time PCR Detection System (Bio-Rad). Results are represented as percentage of input or fold of enrichment, where each antibody signal is normalized to its respective input and then normalized to the nonimmune IgG control signal.

### Western blotting

Whole cell extracts were prepared in radioimmunoprecipitation assay lysis buffer with complete protease inhibitor tablets (54937500; Roche Applied Science). Cell lysates were sonicated on ice at 10% frequency for four cycles (3 s sonication, 10 s between each cycle). Lysates were further passed through a 27 ½-gauge needle with a 1 ml syringe and centrifuged at 15,000*g* for 10 min, and the supernatants were collected. Protein concentration was determined using a Pierce BCA Protein Assay Kit (23227; Thermo Fisher Scientific), and equal amounts of protein (25–70 μg/lane) were separated by electrophoresis using a 4 to 20% polyacrylamide protein gradient gel. After transferring to polyvinylidene difluoride membrane, blots were blocked in PBS supplement with Tween (1%) and bovine serum albumin (5%). Membranes were incubated overnight with the following antihuman primary antibodies: rabbit polyclonal GLI2 antibody (NB600-874; Novus Biologicals; 1∶1000 dilution) and mouse monoclonal α-Tubulin antibody (T9026; Sigma–Aldrich; 1:1000 dilution). Blots were then incubated with anti-rabbit (ALI3403; Biosource; 1∶5000 dilution) or anti-mouse (A3673; Sigma–Aldrich; 1∶5000 dilution) secondary antibodies conjugated with horseradish peroxidase, and signals were visualized by chemiluminescence (34580; SuperSignal West Pico PLUS Chemiluminescent Substrate, Thermo Fisher Scientific). α-Tubulin was measured to control for equal loading.

### Soluble collagen assay

The total soluble collagen in supernatant was measured using the Sircol collagen assay (S1000; Biocolor). Briefly, at previous concentration, samples were incubated with Sircol dye reagent for 30 min at room temperature rotating. The samples were then centrifuged at 13,000 g for 10 min, and the collagen–dye complex precipitate was washed and solubilized in alkali reagent. The dye concentration was estimated by spectrophotometry at 556 nm. Standard curves were used for sample quantification.

### Patient study

The patient's cohort employed in this article was previously described by Sigafoos *et al.* (55). Briefly, we utilized cases from the Mayo Clinic, recruited from October 1, 2000 to July 1, 2010. Recruitment, consent, and biospecimen collection were approved by our institutional review board and have been described previously (55). All patients completed a risk factor questionnaire at enrollment including self-reported Karnofsky performance score, lifestyle, and family history information. Expression studies were achieved by qPCR as we described previously. Fibrosis was assessed by two pathologists with expertise in PC. Slides were evaluated for the amount of visible collagen and were scored according to the pathologists' criteria to a semiquantitative scale (F0–F3).

### CAF infiltration estimation

The MCP-counter algorithm (18) was employed to estimate the CAF abundances in the bulk-RNA-Seq datasets. We downloaded the R package MCP-counter from GitHub and computed the CAF abundances for each sample using the aforementioned algorithm documentation.

### Collagen treatment

PANC-1 cells were seeded into 6-well plates in triplicate. Starvation was done incubating the cells overnight with DMEM and 1% FBS. The media were changed to DMEM and 5% FBS supplemented with 50 μg/ml of type I collagen. RNA was collected 3 h and 24 h, quality control was assayed, and RNA was employed for further sequencing procedure.

### SN38 treatment

A total of 5000 cell/well were seeded into 96-multiwell plates. Next day, cells received pretreatment with type I collagen 50 μg/ml (C3867; Sigma) or vehicle (10 nM acetic acid) overnight. Cells were incubated 24 h with 4 μM SN38 (S4908; Selleckchem) or DMSO in combination with type I collagen or vehicle. Next, viability was measured employing MTT assay.

### MTT assay

PANC-1 or MIA Paca-2 cells were incubated 3 h with a solution of 5 mg/ml of MTT reagent (M5655-1G; Sigma). The insoluble formazan crystals were solubilized incubating with DMSO at room temperature for 5 min. Absorbance was measured using a SpectraMax M3 instrument at a wavelength of 570 nm.

### Cell counting assay

The MIA PaCa-2 cells were seeded into 96-well plates at a density of 5000 cells per well. Next following day, cells were pretreated overnight with type I (50 mg/ml) or vehicle control. After pretreatment, cells were incubated with either SN38 (4 mM) or DMSO (control) in combination with either type I collagen (50 mg/ml) or vehicle for 24 h. The cell number was counted using Celligo Image Cytometer. The percentage of cell increase respect to the initial time point (*t* = 0 h) was calculated.

### RNA-Seq

RNA was prepared from PANC-1 cells according to the Illumina RNA-Seq protocol. cDNA fragments were amplified by PCR and sequenced at both ends using an Illumina Genome Analyzer. Data were aligned to the HG19 reference genome using MAPRseq, v.2.0. The samples passed the manual secondary quality control process, and all samples had over ∼80 million reds and over 97% of the reads map to the human genome. Counts were obtained using htseq-count. Differential expression analysis was achieved using DESeq2. GSEA was completed using GSEA 4.2.3. Gene Ontology analysis was completed using g:Profiler. Visualization was done using Complexheatmap, Enhanced Volcano, ggplot2, and GOplot in R Studio (2023.12.1).

### Expression analysis of public datasets

For RNA-Seq analysis data from The Cancer Genome Atlas (Pan-Cancer atlas, 2018) was obtained through cBioportal. In addition, microarray dataset of 90 primary PC tumors from the International Cancer Genome Consortium has been published (19). RNA-Seq data from 140 primary tumors were previously published and available on cBioPortal (20). MCP-counter v1.2.0 was used to estimate the relative stromal abundance across the datasets and generate CAF scores for tumor samples. As the distributions were largely bimodal, the relative local minima between the distributions were used to separate low and high CAF scores across the datasets. Heatmaps were generated using the R package pheatmap. Tumor samples were ranked by CAF scores for heatmap visualization.

snRNA-Seq data have been published and publicly available from the Broad Institute Single Cell Portal (21). The treatment-naive transcriptomic dataset was used in this study. Transcriptional expression of targeted genes across different cell types was analyzed and visualized with Seurat v5.1.0. The composite expression score for a curated set of 15 matrisome gene markers (*COL11A1*, *COL1A1*, *COL1A2*, *LGALS1*, *LGALS3*, *SPARC*, *PRG2*, *LOXL1*, *TGM2*, *SERPINE1*, SERPINEB3, *PLAU*, *POSTN*, *CXCL12*, and *IL6*) across the snRNA-Seq dataset was generated with addModuleScore function implemented in Seurat.

### Statistical analysis

Evaluation of significance between two groups was done employing Student's *t* test or Mann–Whitney test in the case of nonparametric data. Difference between three or more groups was tested by one-way ANOVA with Dunnet *post hoc* test. Statistical analysis was done using GraphPad Prism 9 software (GraphPad Software, Inc). All the results are expressed as mean ± SD of at least three independent experiments, and *p* < 0.05 was considered statistically significant. References of figures: ∗*p* < 0.05; ∗∗*p* < 0.01; ∗∗∗*p* < 0.001; and ∗∗∗∗*p* < 0.0001.

## Data availability

Raw data from RNA-Seq generated by our laboratory for this paper have been deposited in the Gene Expression Omnibus under the accession number GSE283407.

In addition, Bulk RNA-Seq and clinical data were analyzed. These data are published, and they are accessible through cBioportal (https://www.cbioportal.org/study/summary?id=paad_tcga_pan_can_atlas_2018/). Microarray dataset has been published (19), and it is deposited at the Gene Expression Omnibus under the accession number GSE36924. Additional RNA-Seq expression matrix was analyzed, and it is publicly available (20) at the Genomic Data Commons (https://gdc.cancer.gov/). snRNA-Seq has been published (21) and publicly available from the Broad Institute Single Cell Portal (https://duos.broadinstitute.org/https://duos.broadinstitute.org/) under dataset ID 000139.

## Supporting information

This article contains supporting information.

## Conflict of interest

The authors declare that they have no conflicts of interest with the contents of this article.

## References

[1] Rahib L., Wehner M.R., Matrisian L.M., Nead K.T. (2021). Estimated projection of US cancer incidence and death to 2040. JAMA Netw. Open.

[2] Burris H.A., Moore M.J., Andersen J., Green M.R., Rothenberg M.L., Modiano M.R. (1997). Improvements in survival and clinical benefit with gemcitabine as first-line therapy for patients with advanced pancreas cancer: a randomized trial. J. Clin. Oncol..

[3] Conroy T., Desseigne F., Ychou M., Bouche O., Guimbaud R., Becouarn Y. (2011). FOLFIRINOX versus gemcitabine for metastatic pancreatic cancer. N. Engl. J. Med..

[4] Von Hoff D.D., Ervin T., Arena F.P., Chiorean E.G., Infante J., Moore M. (2013). Increased survival in pancreatic cancer with nab-paclitaxel plus gemcitabine. N. Engl. J. Med..

[5] Siegel R.L., Miller K.D., Fuchs H.E., Jemal A. (2022). Cancer statistics, 2022. CA Cancer J. Clin..

[6] Sherman M.H., Beatty G.L. (2023). Tumor microenvironment in pancreatic cancer pathogenesis and therapeutic resistance. Annu. Rev. Pathol..

[7] Connor A.A., Gallinger S. (2022). Pancreatic cancer evolution and heterogeneity: integrating omics and clinical data. Nat. Rev. Cancer.

[8] Friess H., Yamanaka Y., Buchler M., Ebert M., Beger H.G., Gold L.I. (1993). Enhanced expression of transforming growth factor beta isoforms in pancreatic cancer correlates with decreased survival. Gastroenterology.

[9] Principe D.R., Doll J.A., Bauer J., Jung B., Munshi H.G., Bartholin L. (2014). TGF-beta: duality of function between tumor prevention and carcinogenesis. J. Natl. Cancer Inst..

[10] Horn L.A., Chariou P.L., Gameiro S.R., Qin H., Iida M., Fousek K. (2022). Remodeling the tumor microenvironment via blockade of LAIR-1 and TGF-beta signaling enables PD-L1-mediated tumor eradication. J. Clin. Invest.

[11] Pickup M., Novitskiy S., Moses H.L. (2013). The roles of TGFbeta in the tumour microenvironment. Nat. Rev. Cancer.

[12] Fu Y., Yao N., Ding D., Zhang X., Liu H., Ma L. (2020). TMEM158 promotes pancreatic cancer aggressiveness by activation of TGFbeta1 and PI3K/AKT signaling pathway. J. Cell Physiol.

[13] Akhmetshina A., Palumbo K., Dees C., Bergmann C., Venalis P., Zerr P. (2012). Activation of canonical Wnt signalling is required for TGF-beta-mediated fibrosis. Nat. Commun..

[14] Zhang Q., An Z.Y., Jiang W., Jin W.L., He X.Y. (2023). Collagen code in tumor microenvironment: functions, molecular mechanisms, and therapeutic implications. Biomed. Pharmacother..

[15] Ashina S., Masuda A., Yamakawa K., Hamada T., Tsujimae M., Tanaka T. (2023). A comprehensive analysis of tumor-stromal collagen in relation to pathological, molecular, and immune characteristics and patient survival in pancreatic ductal adenocarcinoma. J. Gastroenterol..

[16] Olivares O., Mayers J.R., Gouirand V., Torrence M.E., Gicquel T., Borge L. (2017). Collagen-derived proline promotes pancreatic ductal adenocarcinoma cell survival under nutrient limited conditions. Nat. Commun..

[17] Linder S., Castanos-Velez E., von Rosen A., Biberfeld P. (2001). Immunohistochemical expression of extracellular matrix proteins and adhesion molecules in pancreatic carcinoma. Hepatogastroenterology.

[18] Becht E., Giraldo N.A., Lacroix L., Buttard B., Elarouci N., Petitprez F. (2016). Estimating the population abundance of tissue-infiltrating immune and stromal cell populations using gene expression. Genome Biol..

[19] Biankin A.V., Waddell N., Kassahn K.S., Gingras M.C., Muthuswamy L.B., Johns A.L. (2012). Pancreatic cancer genomes reveal aberrations in axon guidance pathway genes. Nature.

[20] Cao L., Huang C., Cui Zhou D., Hu Y., Lih T.M., Savage S.R. (2021). Proteogenomic characterization of pancreatic ductal adenocarcinoma. Cell.

[21] Hwang W.L., Jagadeesh K.A., Guo J.A., Hoffman H.I., Yadollahpour P., Reeves J.W. (2022). Single-nucleus and spatial transcriptome profiling of pancreatic cancer identifies multicellular dynamics associated with neoadjuvant treatment. Nat. Genet..

[22] Javanshir H.T., Malekraeisi M.A., Ebrahimi S.S.S., Bereimipour A., Kashani S.F., Bostaki A.A. (2022). Investigation of key signaling pathways and appropriate diagnostic biomarkers selection between non-invasive to invasive stages in pancreatic cancer: a computational observation J. Med. Life.

[23] Tian C., Clauser K.R., Ohlund D., Rickelt S., Huang Y., Gupta M. (2019). Proteomic analyses of ECM during pancreatic ductal adenocarcinoma progression reveal different contributions by tumor and stromal cells. Proc. Natl. Acad. Sci. U. S. A.

[24] Akula S.M., Ruvolo P.P., McCubrey J.A. (2020). TP53/miR-34a-associated signaling targets SERPINE1 expression in human pancreatic cancer. Aging (Albany NY).

[25] Stylianou A., Gkretsi V., Stylianopoulos T. (2018). Transforming growth factor-beta modulates pancreatic cancer associated fibroblasts cell shape, stiffness and invasion. Biochim. Biophys. Acta Gen. Subj..

[26] Wei L., Lin Q., Lu Y., Li G., Huang L., Fu Z. (2021). Cancer-associated fibroblasts-mediated ATF4 expression promotes malignancy and gemcitabine resistance in pancreatic cancer via the TGF-beta1/SMAD2/3 pathway and ABCC1 transactivation. Cell Death Dis..

[27] Xiao Z., Li J., Yu Q., Zhou T., Duan J., Yang Z. (2021). An inflammatory response related gene signature associated with survival outcome and gemcitabine response in patients with pancreatic ductal adenocarcinoma. Front Pharmacol..

[28] Wang Y., Qin C., Zhao B., Li Z., Li T., Yang X. (2023). EGR1 induces EMT in pancreatic cancer via a P300/SNAI2 pathway. J. Transl Med..

[29] Bhatia R., Bhyravbhatla N., Kisling A., Li X., Batra S.K., Kumar S. (2022). Cytokines chattering in pancreatic ductal adenocarcinoma tumor microenvironment. Semin. Cancer Biol..

[30] Yang D., Liu J., Qian H., Zhuang Q. (2023). Cancer-associated fibroblasts: from basic science to anticancer therapy. Exp. Mol. Med..

[31] von Ahrens D., Bhagat T.D., Nagrath D., Maitra A., Verma A. (2017). The role of stromal cancer-associated fibroblasts in pancreatic cancer. J. Hematol. Oncol..

[32] Belhabib I., Zaghdoudi S., Lac C., Bousquet C., Jean C. (2021). Extracellular matrices and cancer-associated fibroblasts: targets for cancer diagnosis and therapy?. Cancers (Basel).

[33] Thorlacius-Ussing J., Jensen C., Nissen N.I., Cox T.R., Kalluri R., Karsdal M. (2024). The collagen landscape in cancer: profiling collagens in tumors and in circulation reveals novel markers of cancer-associated fibroblast subtypes. J. Pathol..

[34] Maneshi P., Mason J., Dongre M., Ohlund D. (2021). Targeting tumor-stromal interactions in pancreatic cancer: impact of collagens and mechanical traits. Front Cell Dev. Biol..

[35] Drifka C.R., Loeffler A.G., Mathewson K., Keikhosravi A., Eickhoff J.C., Liu Y. (2016). Highly aligned stromal collagen is a negative prognostic factor following pancreatic ductal adenocarcinoma resection. Oncotarget.

[36] Tavares-Valente D., Cannone S., Greco M.R., Carvalho T.M.A., Baltazar F., Queiros O. (2023). Extracellular matrix collagen I differentially regulates the metabolic plasticity of pancreatic ductal adenocarcinoma parenchymal cell and cancer stem. Cell Cancers (Basel).

[37] Shields M.A., Dangi-Garimella S., Redig A.J., Munshi H.G. (2012). Biochemical role of the collagen-rich tumour microenvironment in pancreatic cancer progression. Biochem. J..

[38] Perez V.M., Kearney J.F., Yeh J.J. (2021). The pdac extracellular matrix: a review of the ECM protein composition, tumor cell interaction, and therapeutic strategies. Front Oncol..

[39] Masugi Y. (2022). The desmoplastic stroma of pancreatic cancer: multilayered levels of heterogeneity, clinical significance, and therapeutic opportunities. Cancers (Basel).

[40] Garcia P.E., Adoumie M., Kim E.C., Zhang Y., Scales M.K., El-Tawil Y.S. (2020). Differential contribution of pancreatic fibroblast subsets to the pancreatic cancer stroma cell. Mol. Gastroenterol. Hepatol..

[41] Steele N.G., Biffi G., Kemp S.B., Zhang Y., Drouillard D., Syu L. (2021). Inhibition of hedgehog signaling alters fibroblast composition in pancreatic cancer. Clin. Cancer Res..

[42] Vera R.E., Lamberti M.J., Almada L.L., Tolosa E.J., Vrabel A.M., Sigafoos A.N. (2023). GLI1 interaction with p300 modulates SDF1 expression in cancer-associated fibroblasts to promote pancreatic cancer cells migration. Biochem. J..

[43] di Miceli N., Baioni C., Barbieri L., Danielli D., Sala E., Salvioni L. (2024). TGF-Beta signaling loop in pancreatic ductal adenocarcinoma activates fibroblasts and increases tumor cell aggressiveness. Cancers (Basel).

[44] Luo Q., Hu Z., Zhao H., Fan Y., Tu X., Wang Y. (2023). The role of TGF-beta in the tumor microenvironment of pancreatic cancer. Genes Dis..

[45] Mucciolo G., Araos Henriquez J., Jihad M., Pinto Teles S., Manansala J.S., Li W. (2024). EGFR-activated myofibroblasts promote metastasis of pancreatic cancer. Cancer Cell.

[46] Nan P., Dong X., Bai X., Lu H., Liu F., Sun Y. (2022). Tumor-stroma TGF-beta1-THBS2 feedback circuit drives pancreatic ductal adenocarcinoma progression via integrin alpha(v)beta(3)/CD36-mediated activation of the MAPK pathway. Cancer Lett..

[47] Bellone G., Smirne C., Mauri F.A., Tonel E., Carbone A., Buffolino A. (2006). Cytokine expression profile in human pancreatic carcinoma cells and in surgical specimens: implications for survival. Cancer Immunol. Immunother..

[48] Kruger D., Yako Y.Y., Devar J., Lahoud N., Smith M. (2019). Inflammatory cytokines and combined biomarker panels in pancreatic ductal adenocarcinoma: enhancing diagnostic accuracy. PLoS One.

[49] Dayyani F., Macarulla T., Johnson A., Wainberg Z.A. (2023). Second-line treatment options for patients with metastatic pancreatic ductal adenocarcinoma: a systematic literature review. Cancer Treat Rev..

[50] Gupta A., De Jesus-Acosta A., Zheng L., Lee V., Kamel I., Le D. (2023). Clinical outcomes of liposomal irinotecan in advanced pancreatic adenocarcinoma patients previously treated with conventional irinotecan-based chemotherapy: a real-world study. Front Oncol..

[51] Wang W.B., Yang Y., Zhao Y.P., Zhang T.P., Liao Q., Shu H. (2014). Recent studies of 5-fluorouracil resistance in pancreatic cancer World. J. Gastroenterol..

[52] Smolarz B., Durczynski A., Romanowicz H., Hogendorf P. (2021). The Role of microRNA in Pancreatic Cancer. Biomedicines.

[53] Nambaru P.K., Hubner T., Kock K., Mews S., Grube M., Payen L. (2011). Drug efflux transporter multidrug resistance-associated protein 5 affects sensitivity of pancreatic cancer cell lines to the nucleoside anticancer drug 5-fluorouracil. Drug Metab. Dispos..

[54] Muniz V.P., Askeland R.W., Zhang X., Reed S.M., Tompkins V.S., Hagen J. (2013). RABL6A promotes oxaliplatin resistance in tumor cells and is a new marker of survival for resected pancreatic ductal adenocarcinoma patients. Genes Cancer.

[55] Sigafoos A.N., Tolosa E.J., Carr R.M., Fernandez-Barrena M.G., Almada L.L., Pease D.R. (2024). KRAS promotes GLI2-dependent transcription during pancreatic carcinogenesis. Cancer Res. Commun..

