# Supplementary material for: Paracrine regulation of pancreatic cancer cell response to chemotherapy by GLI2–collagen I signaling
Source: J Biol Chem. 2025 May 29;301(7):110311. doi: 10.1016/j.jbc.2025.110311 (PMC12256325; doi:10.1016/j.jbc.2025.110311)
Supplement: Supplementary Figures and Tables [file mmc1.pdf]

Supporting Information

Paracrine regulation of pancreatic cancer cell response to chemotherapy by GLI2-Collagen I signaling

Renzo E. Vera[1], Maite G. Fernandez-Barrena[1], Jose M. Falero[1], John Y. Kwon[1] ,Roberto A. Garza[1], Matthew D. Ross[1], Merih Deniz Toruner[1], Murat Toruner[1], Ezequiel J. Tolosa[1], Luciana L. Almada[1], Huocong Huang[2], Rolf A. Brekken[3], Martín E. Fernandez-Zapico[1]

Material included:

Supplementary figure 1 (page S-1)
Supplementary figure 2 (page S-2)
Supplementary figure 3 (page S-3)
Supplementary figure 4 (page S-4)
Supplementary table 1 (page S-5)

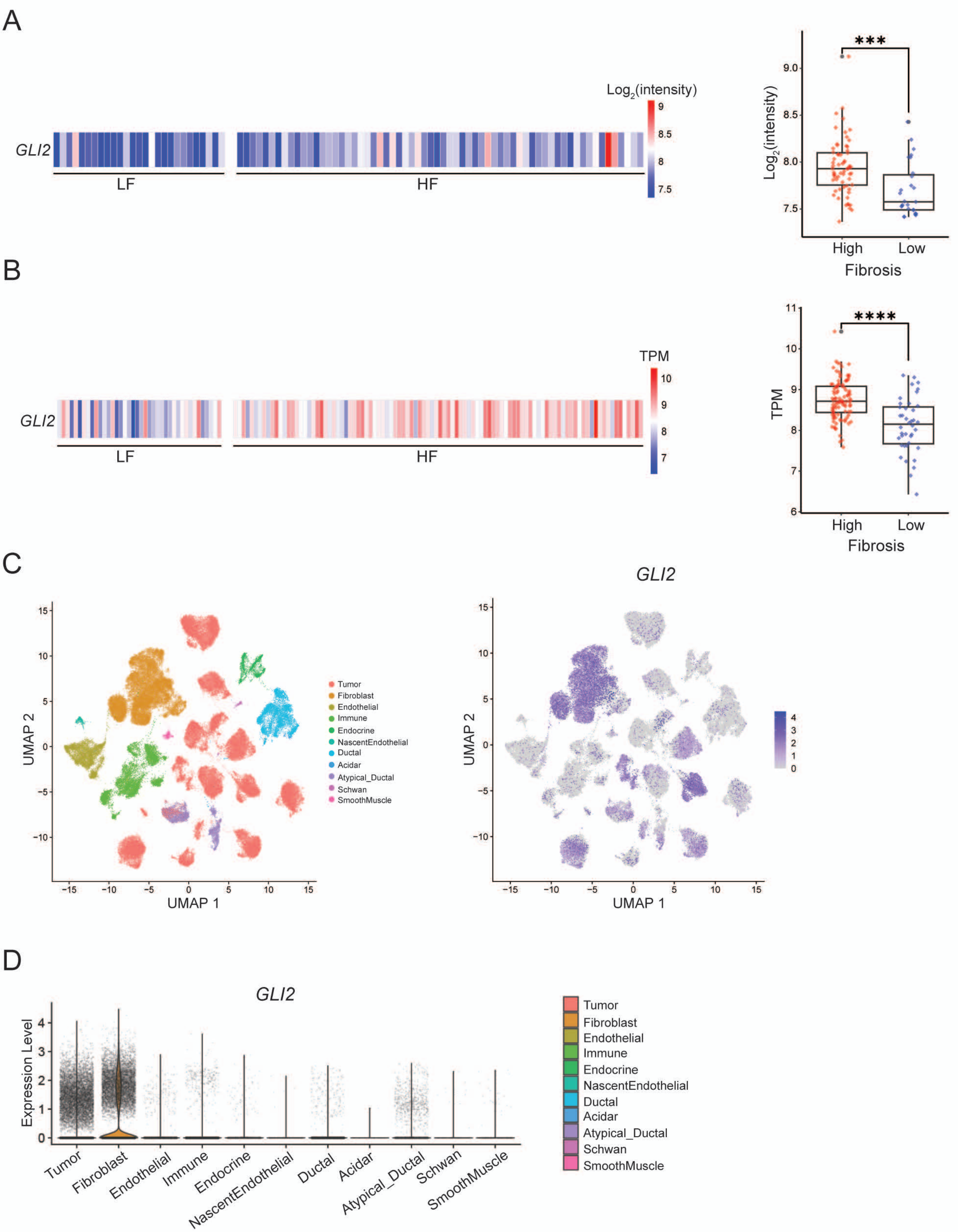

Supplemental Figure 1. *GLI2* expression is associated with the fibroblast compartment in the TME. A) Left, heatmap showing the log2(intensity) for *GLI2* in PC samples (n=91) from publicly available dataset. Samples are ranked by fibrosis scores using MCP-counter and segregated as Low (LF) or High (HF) fibrosis. Right, Boxplots representing the log2(intensity) in both tumor sample groups. B) Left, heatmap showing transcripts per kilobase million (TPM) for *GLI2* in patients (n=140) classified with high or low fibrosis according to MCP-counter from bulk RNA-seq data. Right, Boxplots representing the TPM in both patient groups. C) Left, UMAP plot from snRNA-Seq analysis for PC patient tissues (n=18). Right, UMAP plot shows the expression of the *GLI2* gene in different tumor compartments. D) Violin plots from snRNA-seq showing the distribution of *GLI2* expression level across the different tumor compartments. Mann-Whitney U test was used to assess significance. P<0.001 = ***; P<0.0001 = ****.

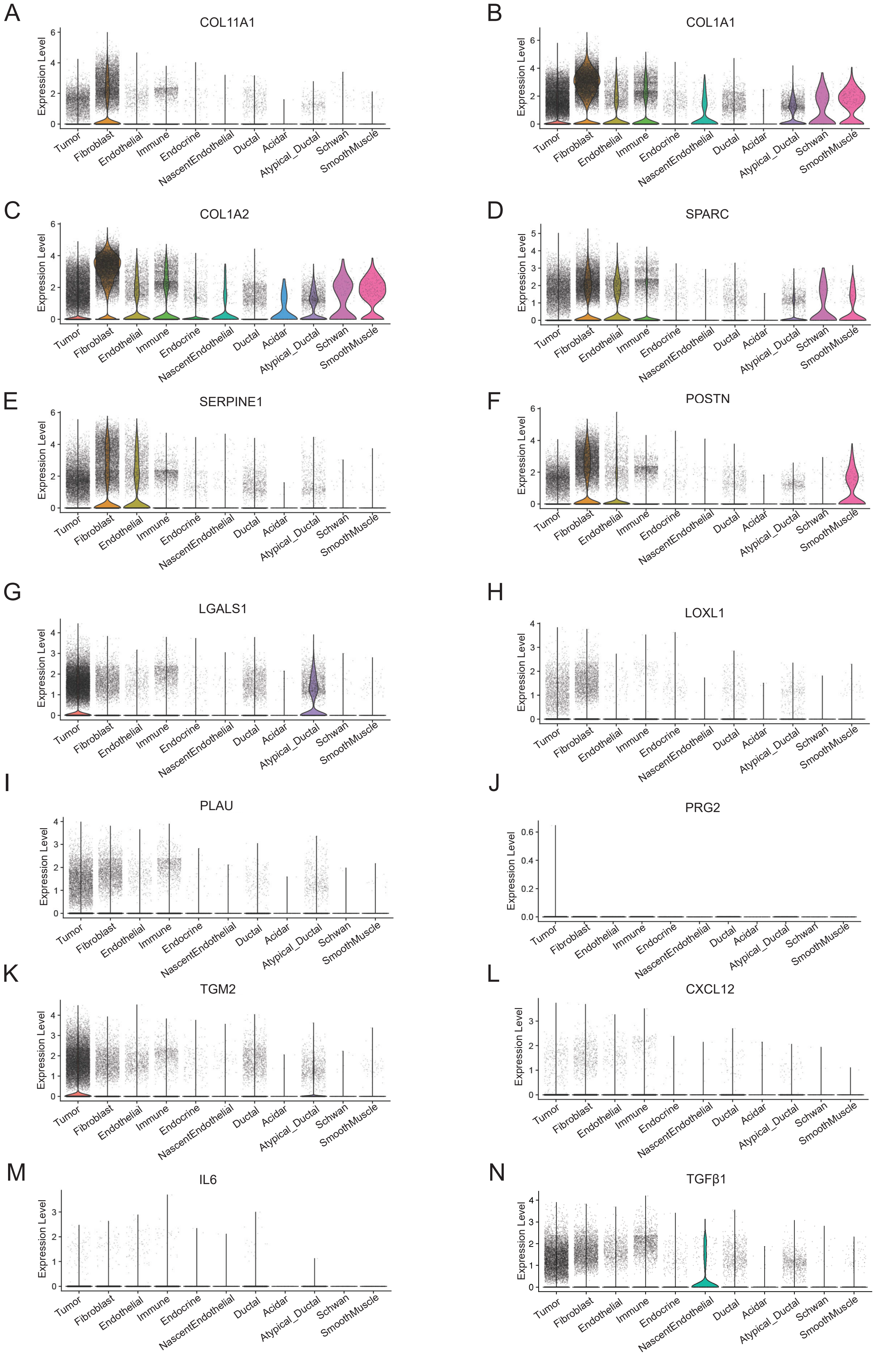

Supplemental Figure 2. *GLI2* is modulating the expression of matrisome genes. Violin plots from snRNA-Seq showing the expression distribution of the top 13 matrisome genes: A) *COL11A1*, B) *COL1A1*, C) *COL1A2*, D) *SPARC*, E) *SERPINE1,* F) *POSTN*, G) *LGALS1*, H) *LOXL1*, I) *PLAU,* J) *PRG2*, K) *TGM2,* L) *CXCL12*, M) *IL6,* N) *TGFβ1*.

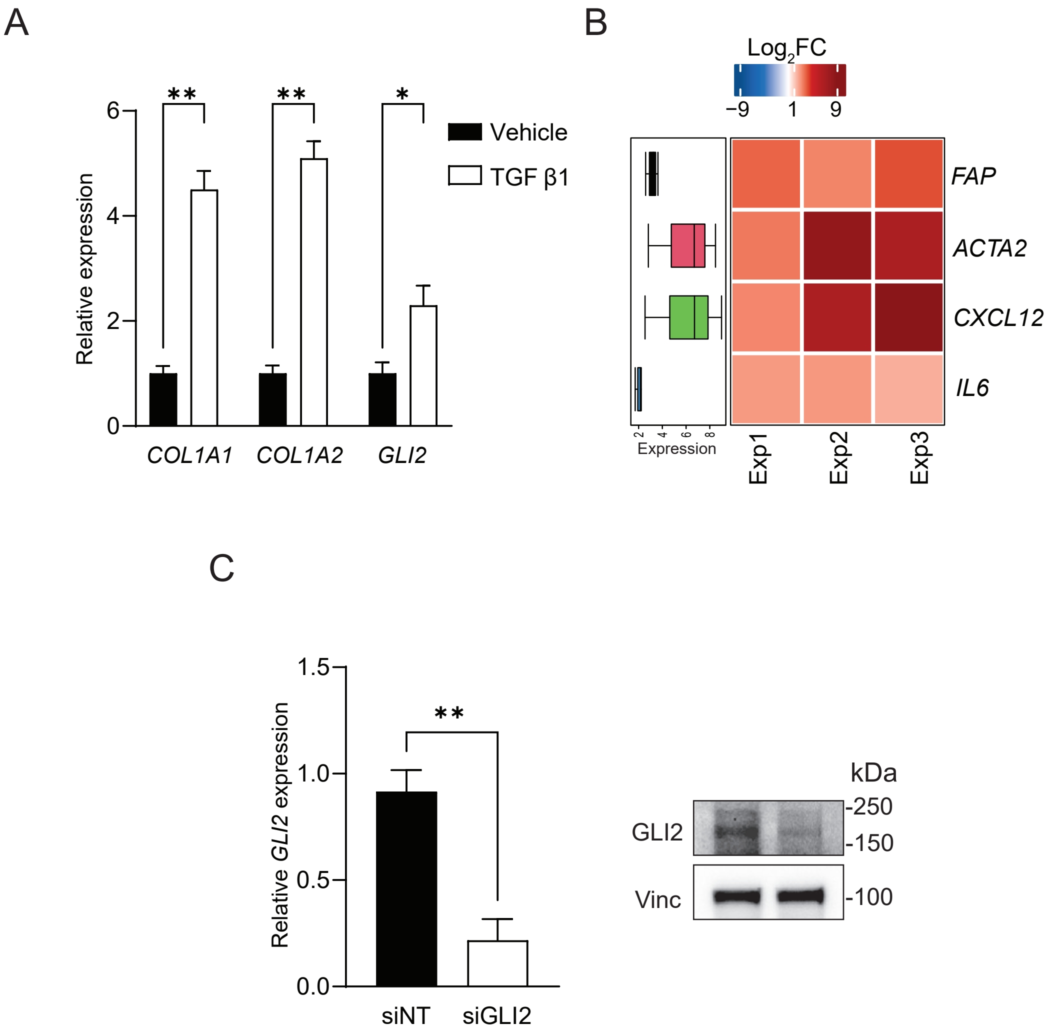

Supplemental Figure 3. TGFβ1 and GLI2 modulate the expression of Collagen I. A) *COL1A1*, *COL1A2,* and *GLI2* relative expression in MRC-5 fibroblasts after 24h treatment with 2 ng/ml TGFβ1. B) heatmap displaying the expression of CAFs markers (*FAP*, *ACTA2*, *CXCL12*, and *IL6*) under TGFβ1 treatment. After 24h of TGFβ1 incubation, MRC-5 cells were harvested, and RNA was extracted. qPCR was assayed. Boxplots represent the expression distribution across 3 independent experiments. C) Left, qPCR results from the same samples incubated with the siRNA system. Right, Western blot showing the expression of GLI2 in HPSC cells incubated with siGLI2 or siNT (control) over 48h. Vinculin was employed as loading control. Results are expressed as means ± SD. Statistical significance, P<0.05 = *; P<0.01 = **.

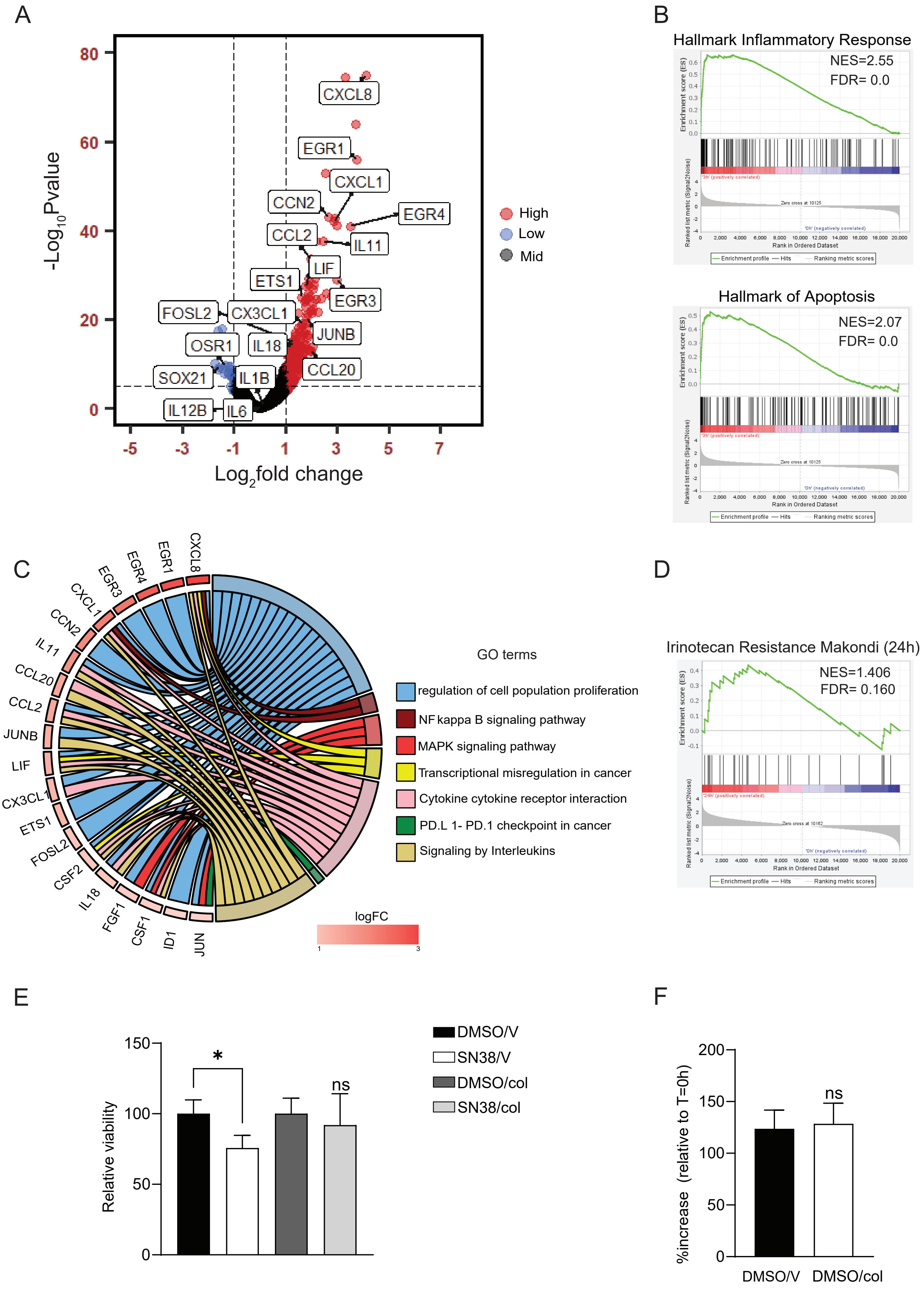

Supplemental Figure 4. Collagen I triggers irinotecan resistance and proinflammatory pathways in PC cells. A) Volcano plot showing downregulated and upregulated genes in PANC-1 cells treated 3h with soluble Collagen I. The cut-off value for log2 fold change is >1 and the cut-off for P value is 10e-6. B) GSEA analysis showing enrichment in gene sets associated with hallmark of inflammatory response (upper chart) and apoptosis (lower chart). C) Chord diagram displaying the relationship between genes identified and the functional pathways (GO term) associated. D) GSEA analysis associated with irinotecan resistance in PANC-1 cells treated with Collagen I for 24h. E) Viability analysis of PANC-1 cells. Cell cultures were incubated for 24h with DMSO or 4 mM of SN38 along with either 50 µg/ml of Collagen (col) or vehicle (V). Following treatment, the viability was evaluated by MTT assay (n=4). F) Panel showing control groups for cell counting assay of MIA Paca-2 cells. Cell cultures were incubated 24h with either DMSO or 4 mM of SN38 in presence of 50 µg/ml of Collagen (col) or vehicle (V). The cell number was determined using Celligo Imager (n=3). Results are expressed as means ± SD. Statistical significance, P<0.05 = *.

SYMBOL	RANK IN GENE LIST	RANK METRIC SCORE	RUNNING ES	CORE ENRICHMENT
BIRC3	3	4.031193256	0.24327867	Yes
CCL2	17	3.308135033	0.44239345	Yes
GLIS3	1014	0.985389888	0.45199275	Yes
PLK2	1529	0.798146546	0.4744358	Yes
FGF2	2635	0.576069057	0.45385626	Yes
TNIK	2685	0.568229854	0.4857144	Yes
PLA2G4C	3136	0.510917306	0.49401948	Yes
ARPC4-TTLL3	3642	0.455965877	0.49625045	Yes
CDH15	3720	0.448058963	0.519449	Yes
COPB2	4045	0.416681111	0.52837676	Yes
INPP4B	4805	0.350312978	0.511501	No
ALDH1L1	4939	0.339032263	0.5253099	No
PRKACB	7222	0.178697482	0.4217607	No
ABCC4	7322	0.172548279	0.42721984	No
CAPS2	7624	0.155559033	0.4215318	No
ZDHHC11	7720	0.149314955	0.4257884	No
TFPI	8123	0.122946247	0.41307038	No
MECOM	8976	0.072089903	0.37473398	No
PRSS23	9381	0.044400841	0.35717267	No
ITGBL1	9448	0.040982813	0.35634053	No
SULF2	12429	-0.138726056	0.21540412	No
HS3ST1	13445	-0.200124085	0.17663209	No
MGLL	13868	-0.224132866	0.16902225	No
GNAS	15296	-0.31379813	0.116471216	No
SPARC	18251	-0.582816422	0.003654545	No
SKIDA1	19874	-1.375769973	0.005461585	No

Supplemental Table 1. Gene List from gene set enrichment analysis (GSEA) in PANC-1 cells treated 3h with soluble Collagen I.
